# Mapping the fly nerve cord

**DOI:** 10.7554/eLife.99804

**Published:** 2024-07-09

**Authors:** Anna Seggewisse, Michael Winding

**Affiliations:** 1 The Francis Crick Institute London United Kingdom

**Keywords:** connectomes, ventral nerve cord, electron microscopy, cell type annotations, descending neurons, *D. melanogaster*

## Abstract

The first neuronal wiring diagram of an insect nerve cord, which includes biological information on cell type and organisation, enables further investigation into premotor circuit function.

**Related research article** Cheong HS, Eichler K, Stürner T et al. 2024. Transforming descending input into behavior: The organization of premotor circuits in the *Drosophila* male adult nerve cord connectome. *eLife* **13**:RP96084. doi: 10.7554/eLife.96084.

**Related research article** Takemura S, Hayworth KJ, Huang GB, Januszewski M, Lu Z, Marin EC, Preibisch S, Xu CS et al. 2024. A connectome of the male *Drosophila* ventral nerve cord. *eLife* **13**:RP97769. doi: 10.7554/eLife.97769.

**Related research article** Marin EC et al. 2024. Systematic annotation of a complete adult male *Drosophila* nerve cord connectome reveals principles of functional organisation. *eLife* **13**:RP97766. doi: 10.7554/eLife.97766.

In the brain, countless neurons connect via synapses to form dense, interconnected networks buzzing with electrical and chemical signals. The resulting neuronal activation patterns allow us to think and feel; in many ways, they define our individual traits and unique quirks.

Such complex computations usually require neural networks that span several subregions of the nervous system. While great advances have been made in investigating neuronal processes in individual subregions, studying the system as a whole has remained difficult. This, in turn, has hindered our ability to understand how specific neural connections give rise to behaviours, let alone individual differences in behaviour between animals.

A tool that reveals global connectivity information is volume electron microscopy. It can be used to produce detailed wiring diagrams that capture how individual neurons are connected, which helps us to understand the functional role of each cell. These ‘connectomes’ provide a comprehensive look at the neural organisation underpinning our behaviour. When linked to genetic tools that allow researchers to record or manipulate neural activity in vivo, they also offer a testbed for experimental studies on how neural circuits work.

Producing connectomes is technically and computationally challenging, and most of the existing ones are of small invertebrates with limited numbers of neurons. The first generation of connectomes included the nematode *Caenorhabditis elegans* (which has about 300 neurons), the sea squirt *Ciona intestinalis* and the annelid *Platynereis dumerilii* (Cook et al., 2019; White et al., 1986; Verasztó et al., 2020; Ryan et al., 2016).

Recent technical advances have made it possible to map out the nervous system of larger organisms – notably insects – as well as the smaller regions of vertebrate brains. The first insect connectomes were of the fruit fly *Drosophila melanogaster*. They included the brain of both the larva and the adult, as well as a partial reconstruction of the female ventral nerve cord (or VNC), a structure akin to the vertebrate spinal cord that integrates sensory information and coordinates motor output (Winding et al., 2023; Dorkenwald et al., 2023; Scheffer et al., 2020; Phelps et al., 2021).

Now, in eLife, Gwyneth Card, Gregory Jefferis, Stuart Berg and colleagues – including Shin-ya Takemura, Kenneth Hayworth, Gary Huang, Michal Januszewski, Zhiyuan Lu, Elizabeth Marin, Stephan Preibisch, and C Shan Xu as first authors – report having generated the first full connectome of the ventral nerve cord of an adult male fruit fly (Takemura et al., 2024). This represents the largest published connectome to date, as it includes over 23,000 neurons connected by more than 10million pre-synapses, 74million post-synapses, and a total length of 44metres of neuronal arbour.

Two companion studies, also to be published in eLife, supplement this resource with additional biological insights: Card and colleagues – including Han Cheong, Katharina Eichler and Tomke Stürner as first authors – describe how the pathways linking brain centers to motor neurons are organised, while Jefferis and colleagues – with Marin as first author – catalogued and grouped the neurons in the VNC connectome based on cell type (Cheong et al., 2024; Marin et al., 2023).

As well as the connectome itself, the foundational work by Takemura et al. (who are based in various institutes in the United States, the United Kingdom and Switzerland) described the robust pipeline the team built to generate the VNC connectome of a five-day-old adult male fly (Figure 1A). This required combining and adapting the state-of-the-art methodologies necessary to prepare and then image the tissue using electron microscopy, as well as the approaches used to align and assemble the resulting images to generate a comprehensive map of the structure. Finally, advanced machine learning algorithms were adjusted so that neurons and their connections could be identified and traced based on this map.

**Figure 1.**
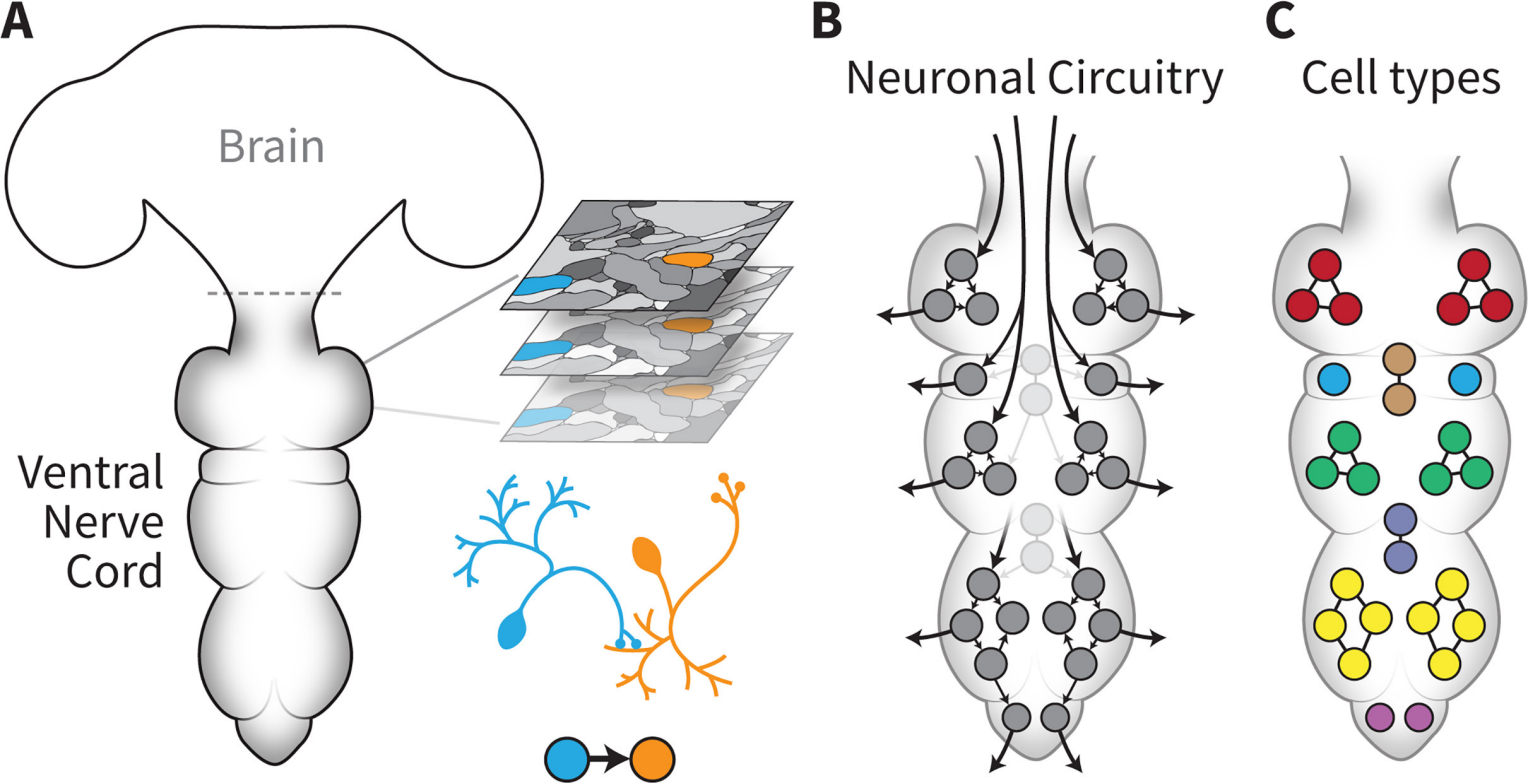
The connectome of the male fruit fly ventral nerve cord. (**A**) Takemura et al. established a pipeline capable of generating a neuronal wiring diagram of the ventral nerve cord (VNC) of a male fruit fly. Volume electron microscopy was used to acquire a large series of images (represented as ‘slices’ on the right) with nanometre resolution across the entire structure. Once these images had been assembled, processed and analysed using automated methods, connections between individual neurons (in orange and blue) could be established and their morphologies reconstructed. (**B**) This connectome provides comprehensive connectivity data (arrows) between all 23,000 neurons (circles) in the VNC, from brain inputs to motor neurons. The work by Cheong et al. focused on using this information to better understand how descending neurons are organised into pathways that help relay information from the brain to the motor neurons that can activate muscles. (**C**) Marin et al. provided additional biological insights into the connectome by classifying all neurons in the VNC into cell types (represented in various colours). This information will help to guide future studies into the role of specific neurons or pathways in shaping various behaviours.

Using the resulting dataset, Cheong et al. examined how descending neurons are organised into pathways within the VNC (Figure 1B). These cells are crucial for movement, as they relay information from the brain to the motor neurons that can activate muscles. Based on these wiring patterns, computational analyses highlighted 37 distinct neuronal communities that may play complementary roles in controlling movements. Certain communities directly connected to motor neurons, with specific pathways linking distinct sets of descending and motor neurons for each leg, for example. Yet others displayed little connectivity with motor neurons; instead, they contained many ‘looping connections’ that might enhance how information circulates within a group of interconnected cells. This may indicate that these pathways coordinate motor control across multiple groups of downstream neurons.

The team also dissected the circuitry underpinning certain types of movement – such as the neural networks that control the leg muscles necessary for walking and turning, or the wing muscles required for steering during a flight. By combining this information with existing experimental findings, many testable hypotheses could be generated to guide further investigation into the precise role of these circuits.

In parallel, Marin et al. classified all neurons by cell type across the VNC connectome. This knowledge allows researchers to bring together cells depending on their connectivity, morphology or gene expression, and to create functional clusters whose role can be explored in subsequent studies. The team systematically categorised each neuron based on their connectivity, and also grouped cells with the same developmental history (such as birth time and lineage). These attributes are associated with neurotransmitter expression, and, in many cases, with the role of the neuron in the circuit. For instance, Marin et al. linked over 5,000 out of the total 6,500 sensory neurons to their respective modality, providing vital information for future studies.

Together, these three bodies of work represent a major advance in neuroscience, describing the first comprehensive map of an insect nerve cord at the synaptic level. The connectivity data, cell type annotations and analysis will be an invaluable resource for future modelling and experimental studies, providing a wealth of hypotheses about how brain signals might be transformed into behaviour.

By agglomerating several state-of-the-art methodologies, the studies by Takemura et al., Cheong et al. and Marin et al. provide an important roadmap to support the generation of other large-scale connectomes. This, in turn, may enable studies aiming to assess how different types of developmental perturbations affect neuronal wiring and behaviour, while also making it possible to establish and then compare the connectomes of specific individuals.

This ‘comparative connectomics’ approach could help reveal whether neuronal connections follow rigid patterns across a population, and if differences in traits and behaviours can be explained by variations in these circuits. For example, comparisons of the current male nerve cord connectome with a future female counterpart might reveal sex-specific wiring differences, in particular related to courtship and mating behaviours. By combining comparative connectomics with in vivo experiments, the goal of determining how changes in nervous system wiring affect behaviour is becoming increasingly tangible.
